# Analyses of the *Dmrt* family in a decapod crab, *Eriocheir sinensis* uncover new facets on the evolution of DM domain genes

**DOI:** 10.3389/fphys.2023.1201846

**Published:** 2023-05-26

**Authors:** Peng Zhang, Yanan Yang, Yuanfeng Xu, Zhaoxia Cui

**Affiliations:** ^1^ School of Marine Sciences, Ningbo University, Ningbo, China; ^2^ Laboratory for Marine Biology and Biotechnology, Pilot Qingdao National Laboratory for Marine Science and Technology (Qingdao), Qingdao, China

**Keywords:** sexual development, *Dmrt*, evolution, malacostraca, *Eriocheir sinensis*

## Abstract

DM domain genes are a group of transcription factors that are integral to sexual development and its evolution in metazoans. Their functions and regulatory mechanisms are not well understood in Malacostraca (crabs and crayfish) while these sex regulators have been widely identified in the past decade. In this study, the *Dmrt* family was investigated in the decapod crab, *Eriocheir sinensis*. We find that most members of the *EsDmrt* family begin to enrich around the juvenile 1 stage. In reproductive organs, *EsDsx1*, *EsDsx2*, *EsiDMY* and *EsiDmrt1a* highly express in the male-specific androgenic gland (AG), while *EsDmrt-like*, *EsDsx-like*, *EsDmrt11E*, and *EsiDmrt1b* show relatively high expression in testis. Also, we find the highly aberrant expression of *EsiDMY* and *EsiDmrt1a* in the chimeric AG, strongly indicating their function in AG development. Moreover, RNA interference of *EsDsx1*, *EsiDMY*, and *EsiDmrt1a* results in a significant decrease in transcription of the *Insulin-like androgenic hormone* (*IAG*), respectively. Our findings suggest that *Dmrt* genes in *E. sinensis* primarily function in male sexual differentiation, especially in AG development. Besides, this study identifies two unique groups of *Dmrt* genes in Malacostraca: *Dsx* and *iDmrt1*. In Malacostraca *Dsx*, we uncover a cryptic mutation in the eight zinc motif-specific residues, which were firmly believed to be invariant across the *Dmrt* family. This mutation sets the Malacostraca *Dsx* apart from all the other *Dmrt* genes and implies a different way of transcriptional regulation. Genes from the *iDmrt1* group show phylogenetical limitation to the malacostracan species and underwent positive selection, suggesting their highly specialized gene function to this class. Based on these findings, we propose that *Dsx* and *iDmrt1* in Malacostraca have developed unique transcriptional regulation mechanisms to facilitate AG development. We hope that this study would contribute to our understandings of sexual development in Malacostraca and provide new insights into the evolutionary history of the *Dmrt* family.

## 1 Introduction

Animals have evolved to become sexual dimorphism in the power of sexual selection (Darwin, 1888). Despite the various sexually dimorphic traits, the upstream regulators that contribute to these discrepancies are highly conserved (Zarkower, 2001). Recent years, the center of these regulators converge on the family of DM domain genes, also known as the *Doublesex and male abnormal-3 related transcription factor* (*Dmrt*) (Volff et al., 2003; Hong et al., 2007; Kopp, 2012). The essence of the *Dmrt* family is a group of transcription factors that contain the DM DNA binding domain, which was firstly identified in the *Drosophila melanogaster Dsx* (Hildreth, 1965) and *Caenorhabditis elegans mab-3* (Shen and Hodgkin, 1988) genes. The discovery of the evolutionary conservation of these two genes in structure and function soon leads to numerous identifications in other species (Raymond et al., 1998).

The *Dmrt* family is widely distributed across the animal kingdom and includes various groups (Wexler et al., 2014; Picard et al., 2015). In vertebrates, the *Dmrt1* gene is essential to sexual development: promoting postnatal testis differentiation in mammalian (Raymond et al., 2000; Matson et al., 2011), maintaining sexual bias in avian (Smith et al., 2009), and preventing sexual transition in fish (Wu et al., 2012). Sometimes, the mutated *Dmrt1* homologue *DM domain on Y* (*DMY*) (Kobayashi et al., 2004) and *DM domain on W* (*DMW*) (Yoshimoto et al., 2008) could take over sex determination due to the amino acid substitution (Ogita et al., 2020).

In invertebrates, the genes of *Dmrt* family are broadly divided into four groups: *Dsx*, *Dmrt11E*, *Dmrt93B* and *Dmrt99B* (Panara et al., 2019). While the functions of some of these genes are not yet well understood, the *Dsx* gene has been found to have some similarities in expression pattern and functionality to the vertebrate *Dmrt1* gene (Matson and Zarkower, 2012). Besides, a unique group of *Dmrt* genes with two DM domains was specifically found in the Malacostraca. Initial research of the group was conducted on *Sagmariasus verreauxi* (Chandler et al., 2017) and soon extended to other species, such as *E. sinensis* (Cui et al., 2021), *Macrobrachium rosenbergii* (Abayed et al., 2019) and *Scylla paramamosain* (Wan et al., 2021).

The *Dmrt* family is a group of transcription factors with a conserved DNA-binding motif known as the DM domain (Raymond et al., 1998). The DM domain consists of two intertwined structures that chelate two atoms of zinc and binds to the minor groove of target gene in transcriptional regulation (Zhu et al., 2000). In Pancrustacea, the *Dmrt11E*, *Dmrt93B*, and *Dmrt99B* genes have been shown to play less important roles in sexual development compared to the *Dsx* gene. (Kato et al., 2011; Panara et al., 2019). In Hexapoda, *Dsx* is alternatively spliced into sex-specific isoforms (Chikami et al., 2022), which control the transcription of the target gene *yolk protein*/*vitellogenin* (Burtis et al., 1991), contributing to the sexual differentiation of both genders. In Branchiopoda, the targeted gene of *Dsx* remains unclear, but a recent transcriptomic analysis suggests a vitellogenin receptor could be the downstream element of *Dsx* (Nong et al., 2020). In Branchiopoda and Malacostraca, *Dsx* transcribes male-specifically, controls the development of male-specific structures, and is dispensable for female sexual differentiation (Wexler et al., 2019). In Malacostraca, the common regulator of male sexual differentiation is *IAG* (Chandler et al., 2018), which is secreted by the class-specific endocrine organ androgenic gland (AG, Ventura et al., 2011). And putative binding sites of *Dmrt* have been found on the promoter of *IAG* in many malacostracan species (Li et al., 2018; Cui et al., 2021; Wan et al., 2022; Wei et al., 2022; Wang et al., 2023). Moreover, homologue of the *vitellogenin* was also found in the *S. paramamosain*, but no *Dmrt* binding site was found on the promoter (Wan et al., 2022).

In this study, we investigated the *Dmrt* family in *E. sinensis* and analyzed its function in AG development. Additionally, we conducted evolutionary analyses of the *Dmrt* genes, reconstructed their ancestral sequences, and proposed a hypothesis for the evolution of the family. The discovery of the Malacostraca-specific *Dsx* and *iDmrt1* groups supports the idea that the *Dmrt* gene family in this class may have undergone a unique evolutionary event that differs from that of Hexapoda and Branchiopoda.

## 2 Materials and methods

### 2.1 Crab sample and RNA preparation

We reasoned that male reproductive tissues were likely to express *Dmrt*, including splicing isoforms of these genes that might exist. To obtain different stages of testis and androgenic gland, crabs were purchased separately in August and October from a local market in Ningbo city (Zhejiang, China). Crabs were anesthetized on ice for 15 min before dissection. Testis at the spermatid stage and AG at the synthesis stage were gently dissected from crabs bought in August, and testis at the sperm stage and AG at the secretion stage were gently dissected from crabs bought in October. The fresh reproductive organs were plunged into TRIzol reagent (Invitrogen, United States) and stored at −80°C for RNA extraction. Total RNA was extracted by grinding tissue in TRIzol reagent (Invitrogen, United States) and processed following the manufacturer’s instructions. RNA quality was guaranteed by NanoOne and 1.2% agarose gel. The integrity of the RNA was determined with the Agilent 2100 Bioanalyzer (Agilent Technologies, United States). Besides, chimeric crabs, collected in local crab farms, are genetically females whereas exhibit male characteristics such as AG and male gonopods (Zhu et al., 2022).

### 2.2 PacBio sequencing

Four SMRT libraries were generated from collected tissues, each corresponding to a specific stage of testis or AG development: T_S2 (testis at the spermatid stage), T_S3 (testis at the sperm stage), AG_S2 (AG at the synthesis stage), and AG_S3 (AG at the secretion stage). The libraries were constructed using the SMRTbell Template Prep Kit from Pacbio (United States). The mRNA was first converted to cDNA using the SMARTer PCR cDNA Synthesis Kit from Takara (United States). The cDNA was then amplified by PCR and purified using AMPure PB beads from Pacbio (United States). Finally, the resulting templates were sequenced on a Sequel II instrument from Pacbio (United States).

### 2.3 Identification and characterization of the *EsDmrt* family

To get a comprehensive understanding of the *EsDmrt* family, we annotated a broad transcriptomic library of *E. sinensis* (annotation details are listed in Supplementary Table S1), spanning from embryonic and larva stages, normal tissues of adult crab, and reproductive organs after eyestalk ablation and *IAG* knockdown (accession numbers of transcriptomes used for annotation are listed in Supplementary Table S2). After annotation, we extracted all clusters that were annotated as DM domain genes in at least one database, serving as a query to conduct BLASTn against the newly published genome (GCA_013436485.1) of *E. sinensis*. The genomic locations of potential *EsDmrt* genes were marked on the general feature format (GFF) file. Then we aligned the merged SMRT library to the genome using Minimap2 v2.17 with the command “minimap2 -ax map-pb.” Corresponding full-length transcripts of *EsDmrt* genes were fetched (name of transcripts are listed in Supplementary Table S3). Gene structures and alternative splicing events were analyzed on IGV using the binary sequence alignment/map (BAM) file obtained from the alignment. ORFs and deduced amino acids were predicted by Expasy Translate (https://web.expasy.org/translate/). Functional domains were predicted by SMART program (http://smart.embl-heidelberg.de/). Genomic structure visualization was performed by GSDS v2.0 (http://gsds.gao-lab.org/). Visualization of chromosome location was conducted by MapChart (Voorrips, 2002). Multiple sequence alignment was conducted by MAFFT v7.407 (Katoh and Standley, 2013) and visualized by GeneDoc (Nicholas, 1997).

### 2.4 Molecular phylogenetic analysis

Phylogenetic analysis of DM domain genes was performed using amino acid sequences of the N-terminal DM domain (*Dmrt* genes used for the analysis are listed in Supplementary Table S4; amino acid sequences used for alignment can be found in Supplementary Material). We aligned the sequences using MAFFT with the L-INS-I option (most accurate model) and built a maximum-likelihood (ML) tree with 1,000 bootstrap values in the IQ-TREE software (Minh et al., 2020). The substitution model was automatically selected using “-MPF” and LG+I+G4 was the best-fit. Finally, the tree was visualized and annotated using GGTREE (Yu et al., 2017).

### 2.5 Gene expression analysis using transcriptomic data

To investigate the function of the *EsDmrt* family in sexual development, we conducted a wide-ranging analysis of the expression levels in gonads, early-stage transcriptomic library (Illumina data) and chimeric tissues (NanoPore data). Illumina data (SRA numbers are listed in Supplementary Table S2) were generated from our previous studies or downloaded from NCBI and then trimmed by Trimmomatic v0.39. The clean data were mapped to the genome with Hisat2 v2.1.0. Consequent BAM and GFF files were used to calculate the transcripts per million (TPM) values by Stringtie v2.1.3. NanoPore data (Unpublished data from our lab) were aligned to the genome using minimap2 v2.17 after quality control and consequent BAM files were used to calculate the TPM values as described above. All TPM values were presented as mean ± SD. Statistical analyses were performed by the one-way analysis of variance (ANOVA) or *T*-test using SPSS 28 software. *p-*values less than 0.05 and 0.01 were considered significant and extremely significant. Each group contained three biological repetitions. The results were visualized by ggplot2 (https://ggplot2.tidyverse.org).

### 2.6 RNAi and RT-qPCR

The siRNA of *EsDsx1* ^*M1*^, *EsDsx1* ^*M2*^, *EsiDmrt1a*, *EsiDMY* ^*L*^ and siControl were chemically synthesized by GenePharma (Shanghai, China) (sequences of the siRNAs and control siRNA are listed in Supplementary Table S5). Crab saline was synthesized in the lab (2.570 g NaCl, 0.084 g KCl, 0.148 g CaCl_2_, 0.248 g MgCl_2_, 0.327 g Na_2_SO_4_, 0.238 g HEPES, pH 7.4, total 100 mL). The siRNA was incubated with GP-transfect for 30 min before injection. The experiment included three treatments, namely, siRNAi, siControl and saline, with each treatment performed in six replicates (*n* = 6). A total of 40 μg/individual siRNA was injected into the fifth walking leg’s sinus. After 24 h of injection, only AG was collected in three treatments. Animal dissection and RNA extraction were conducted in the same way as described above. RT-qPCR was performed to investigate the expression level. *Dmrt* and *IAG* expression relative to the siControl and saline were determined by the 2^−ΔΔCt^ method. The Shapiro-Wilk test and Levene’s test were used to check the normality and homogeneity of variance assumptions, respectively. Independent sample *T*-tests were used to analyze *Dmrt* and *IAG* expression between RNAi and siControl groups. *p-*values less than 0.05 and 0.01 were considered significant and extremely significant. Results of statistical tests are listed in Supplementary Table S6.

### 2.7 Consensus analysis of DM domain

To gain insights into the conservation and variation of the DM domain across different species and groups including *Dmrt1* from various metazoans, four conserved groups from Pancrustacea and *iDmrt1* from Malacostraca, we conducted amino acid sequence alignments and showed their conservation using Weblogo v3.7.4 (http://weblogo.threeplusone.com/). Accession numbers of sequences used for the analysis are listed in Supplementary Table S7.

### 2.8 Positive selection analysis and ancestral sequence reconstruction

The ratio of nonsynonymous (*d*_N_) and synonymous (*d*_S_) substitution rates (*ω* = *d*_N_/*d*_S_) were evaluated using codeML in PAML version 4.9 (Yang, 2007). For the *iDmrt1* group, we used site models that assume different *ω* varies among sites but not branches, including model M0 (one ratio), M1a (neutral), M2a (selection), M3 (discrete), M7 (beta), and M8 (beta and *ω* > 1) (Yang et al., 2000). The reference tree was built first (tree diagram is shown in Supplementary Figure S1; *Dmrt* genes used for the analysis are listed in Supplementary Table S8; nucleotide sequences used for the alignment can be found in Supplementary Material), then pairwise comparisons of M0 versus M3, M1a versus M2a, and M7 versus M8 were used to perform likelihood ratio test (LRT). For the *Dsx* group, we used the branch-site model that assumes *ω* to vary both among sites and branches, to compare the model A against the model Null (fixed *ω* of 1). Also, we built a reference tree first (tree diagram is shown in Supplementary Figure S2; *Dmrt* genes used for the analysis are listed in Supplementary Table S9; nucleotide sequences used for the alignment can be found in Supplementary Material), then the comparison of model A versus model Null was used to perform LRT. Both significance tests were performed using the *χ* ^2^ distribution with degrees of freedom of 2 and 1 for *iDmrt1* and *Dsx* groups, respectively. If the results of LRT showed significance, Bayes empirical Bayes (BEB) analysis would be conducted to identify positively selected sites with a posterior probability greater than 0.95. Meanwhile, ancestral sequences of *Dsx* were reconstructed using the empirical Bayes approach implemented in PAML (Yang et al., 1995).

### 2.9 Protein structure prediction and motif prediction

The reconstructed ancestral sequences of *Dsx* were utilized as inferred protein sequences to model structural domains using the Alphafold2-based algorithm (ColabFold: Mirdita et al., 2022) with the default settings. The accuracy of predicted models was assessed using the predicted Local distance difference test (plDDT) score, which was automatically calculated on the ColabFold platform (diagram of plDDT score is shown in Supplementary Figure S3). The PyMOL Molecular Graphics System, Version 2.0 (Schrödinger, 2015) was used to visualize the 3D models of the predicted structures and we highlighted the zinc motif-specific residues cysteine and histidine with red and yellow, respectively. Then we used the online software ZincExplorer to detect the zinc binding ability of the reconstructed amino acids. Moreover, we modelled the *Dsx* protein from several malacostracan species using Swiss-Model.

## 3 Results

### 3.1 PacBio sequencing of *Eriocheir sinensis*

We constructed four SMRT libraries (T_S2, T_S3, AG_S2 and AG_S3) using testis at spermatid and sperm stages, and AG at synthesis and secretion stages. We obtained a total of 171,436, 348,229, 187,041 and 204,937 circular consensus sequences from raw reads, of which 58,046,178,242,118,195 and 116,436 were identified as full length non-chimeric reads. After clustering, we identified 35,011, 103,219, 54,833 and 68,126 high-quality isoforms with an average length of 1,092, 1,633, 904 and 1,429 bp, respectively. Finally, four libraries were merged, resulting in a total of 197,160 nonredundant full-length transcript. The complete datasets were deposited in the NCBI Sequence Read Archive (SRA) under accession numbers SRR24067590, SRR24067589, SRR24067592 and SRR24067591.

### 3.2 Identification and characterization of *EsDmrt* genes

**FIGURE 1:**
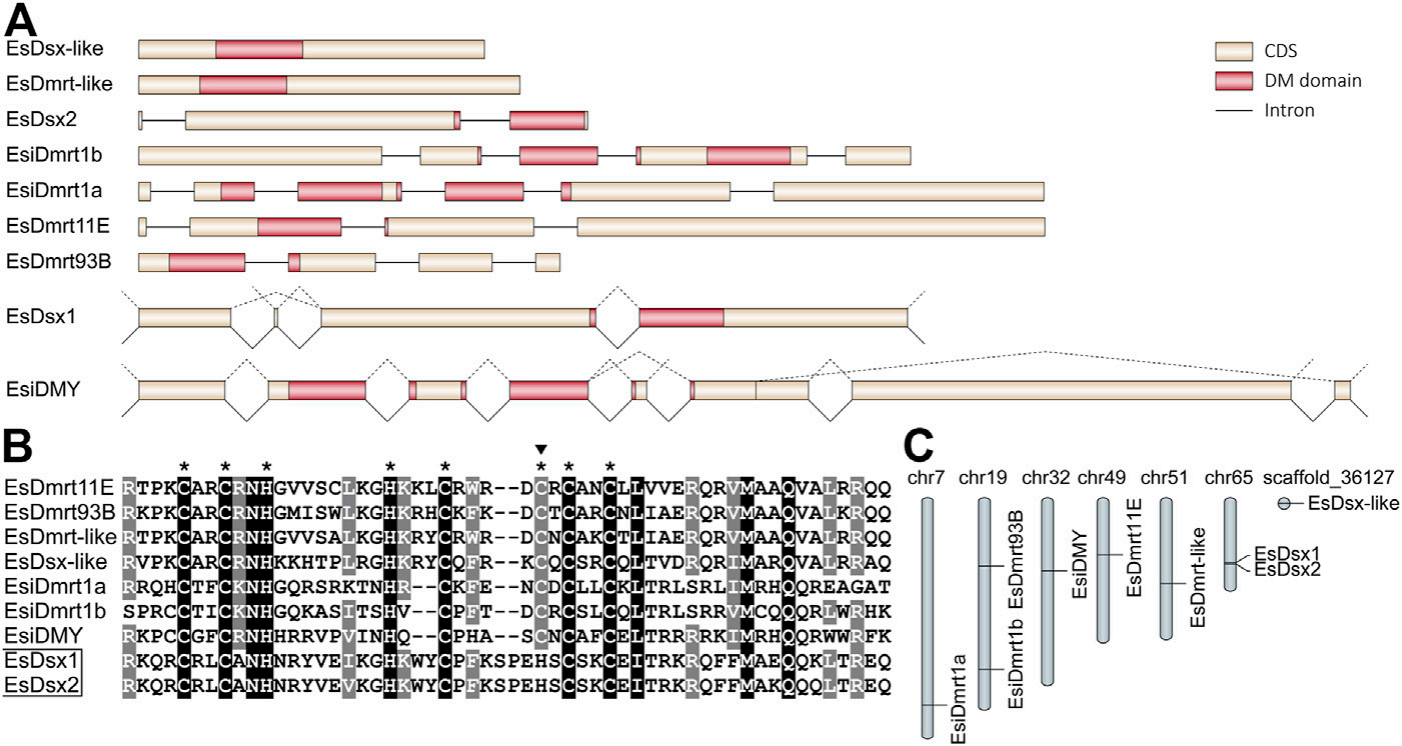
Characterization of *Dmrt* family genes in *E. sinensis*. **(A)** Structure features of nine *EsDmrt* genes. All elements are shown to scale except for introns. The alternative splicing events of *EsDsx1* and *EsiDMY* are displayed above the genes with dotted lines. **(B)** Alignment of deduced amino acid sequences of DM domains (N-terminal domain of *EsiDmrt1a*, *EsiDmrt1b* and *EsiDMY* are used in this alignment). Identical amino acids are highlighted in black and similar ones in grey. Eight conserved motif-specific residues are indicated with asterisks and the point mutation in *EsDsx1* and *EsDsx2* is indicated with a top solid triangle. **(C)** Genome loci of nine *EsDmrt* genes. Except for the *EsDsx-like*, all genes have their clear chromosome locations.

Our study identified nine members of the *Dmrt* family in *E. sinensis*, including *EsDsx1*, *EsDsx2*, *EsDsx-like*, *EsiDmrt1a*, *EsiDmrt1b*, *EsiDMY*, *EsDmrt-like*, *EsDmrt11E* and *EsDmrt93B* (Figure 1A). The nine genes exhibit highly conserved DM domains, except for *EsDsx1* and *EsDsx2*, in which a point mutation was both detected in the zinc motif-specific residues (Figure 1B). *EsDsx1* and *EsiDMY* are alternatively spliced (Figure 1A), and no sex-specific splicing isoforms were detected. While we found two *EsDsx1* isoforms (*EsDsx1* ^*M1*^ and *EsDsx1* ^*M2*^) specifically expressed in AG and one isoform (*EsDsx1* ^*C*^) that was commonly expressed (Supplementary Figure S4). Splicing isoforms of *EsiDMY* encoded three proteins of different lengths (*EsiDMY* ^*S*^, *EsiDMY* ^*M*^ and *EsiDMY* ^*L*^), and their presence was confirmed by RT-PCR (Supplementary Figure S5). Besides, most *EsDmrt* genes were located on a single chromosome, while *EsDsx1* and *EsDsx2* were adjacent to each other (Figure 1C).

### 3.3 Molecular phylogenetic analysis of DM domain genes

**FIGURE 2:**
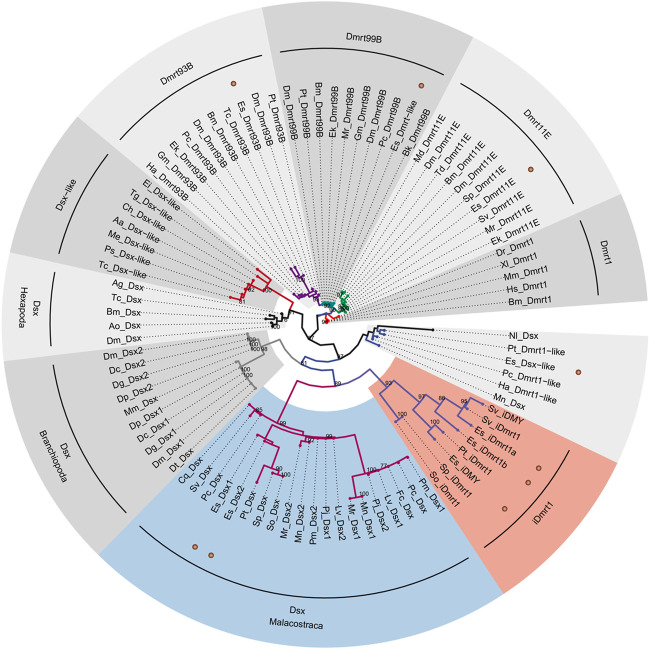
Molecular phylogeny of *Dmrt* family genes in *E. sinensis*. Phylogenetic analysis was based on amino acid sequences of DM domain and performed by IQ-TREE following multiple sequence alignment using MAFFT. The maximum-likelihood method was applied. The 91 taxonomic units conducted for this analysis are listed in Supplementary Table S4. Numerical value on each node indicates bootstrap value that is >70%. *EsDmrt* genes are indicated with brown dots. Malacostraca *Dsx* and *iDmrt1* groups are colored in light steel blue and light coral.

We built a ML tree based on 91 DM domain genes to reconstruct the phylogenetic relationship of the *EsDmrt* family (Figure 2). Our analysis revealed that five *EsDmrt* genes were distributed in four conserved clades of Pancrustacea: *Dsx*, *Dmrt11E*, *Dmrt93B*, and *Dmrt99B*. Notably, the *Dsx* clade was classified into three reliable subclades, including Hexapoda *Dsx* clade, Branchiopoda *Dsx* clade and Malacostraca *Dsx* clade. Besides, we found that three *EsDmrt* genes fall into a monophyletic branch consisting of *iDmrt1* orthorlogues from malacostracan species. The phylogeny of *EsDsx-like* remained unclear in our study.

### 3.4 Expression pattern of *EsDmrt* genes in three transcriptomic libraries

**FIGURE 3:**
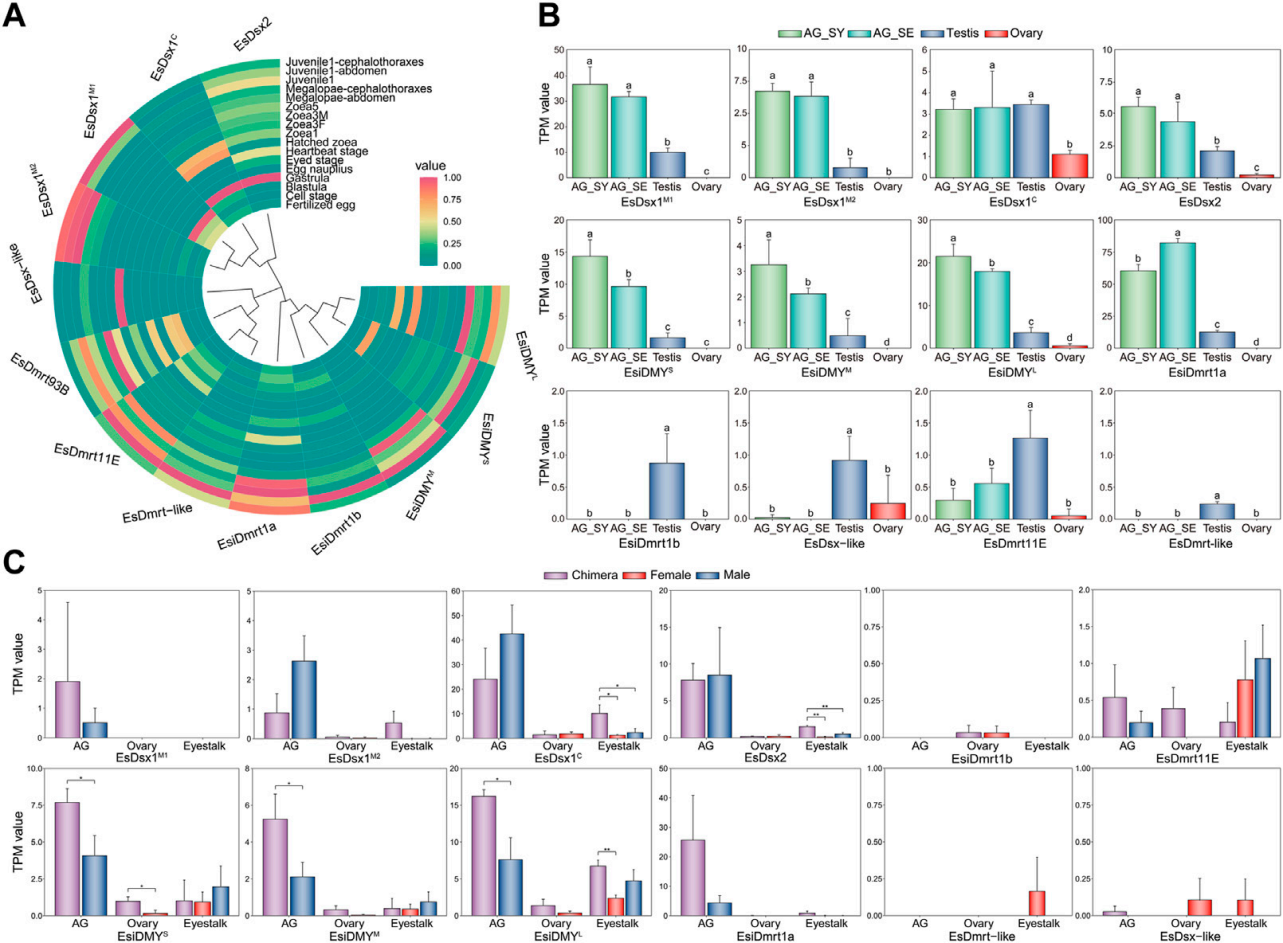
Expression level of the *Dmrt* family genes in three different transcriptomic libraries of *E. sinensis*. **(A)** Expression pattern of *EsDmrt* genes in embryonic, larval and juvenile stages. The heatmap was shown as normalized TPM value. **(B)** Expression pattern of *EsDmrt* genes (including splicing isoforms) in reproductive organs (AG, testis and ovary). The bar plot was shown as TPM value. Vertical bars represented mean ± SD, *n* = 3. Results were analyzed by one-way ANOVA and different letters (a-d) above the bars presented significant differences between groups (*p* < 0.05). AG_SY, androgenic gland at synthesis stage; AG_SE, androgenic gland at secretion stage. **(C)** Expression pattern of the *EsDmrt* genes in normal and chimeric *E. sinensis*. The bar plot was shown as TPM value. Vertical bars represented mean ± SD, *n* = 3. Tissues from left to right: AG-androgenic gland (chimeric, normal male); ovary (chimeric, normal female); eyestalk (chimeric, normal female and male). Tissue and organ from chimeric, normal female and male were colored with purple, red and blue. Vertical bars represented mean ± SD, *n* = 3. Results were analyzed by independent *T*-test comparing chimeric and normal tissue and organ (*p < 0.05, **p < 0.01).

We analyzed expression patterns of the *EsDmrt* family using transcriptomic data from three libraries. The embryonic and larval transcriptomes show that most *EsDmrt* genes start to enrich around the juvenile 1 stage (Figure 3A), while *EsDsx1* ^*M1*^, *EsDsx1* ^*M2*^ and *EsDsx2* show relatively high expression in gastrula and blastula stages. In reproductive organs, *EsDsx1*, *EsDsx2*, *EsiDMY* and *EsiDmrt1a* are co-expressed predominantly in male organs, particularly in two stages of AG, where their expression levels are significantly higher than testis and ovary (Figure 3B, one-way ANOVA, *p* < 0.05). Besides, the overall expression levels of *EsiDmrt1b*, *EsDmrt11E*, *EsDmrt-like* and *EsDsx-like* are relatively low, but they still show a significantly higher expression in testis (One-way ANOVA, *p* < 0.05). The expression level of *EsDmrt93B* is too low to show. In chimeric crabs, we find a significantly higher expression of *EsiDMY* in the chimeric AG compared to the normal ones (Figure 3C, independent sample *T*-test, *p < 0.05). Additionally, *EsDsx1* ^*C*^, *EsDsx2* and *EsiDMY* ^*L*^ show significantly higher expression levels in the chimeric eyestalk compared to the normal ones (independent sample *T*-test, *p < 0.05).

### 3.5 Regulation of *EsDmrt* for *EsIAG* expression

**FIGURE 4:**
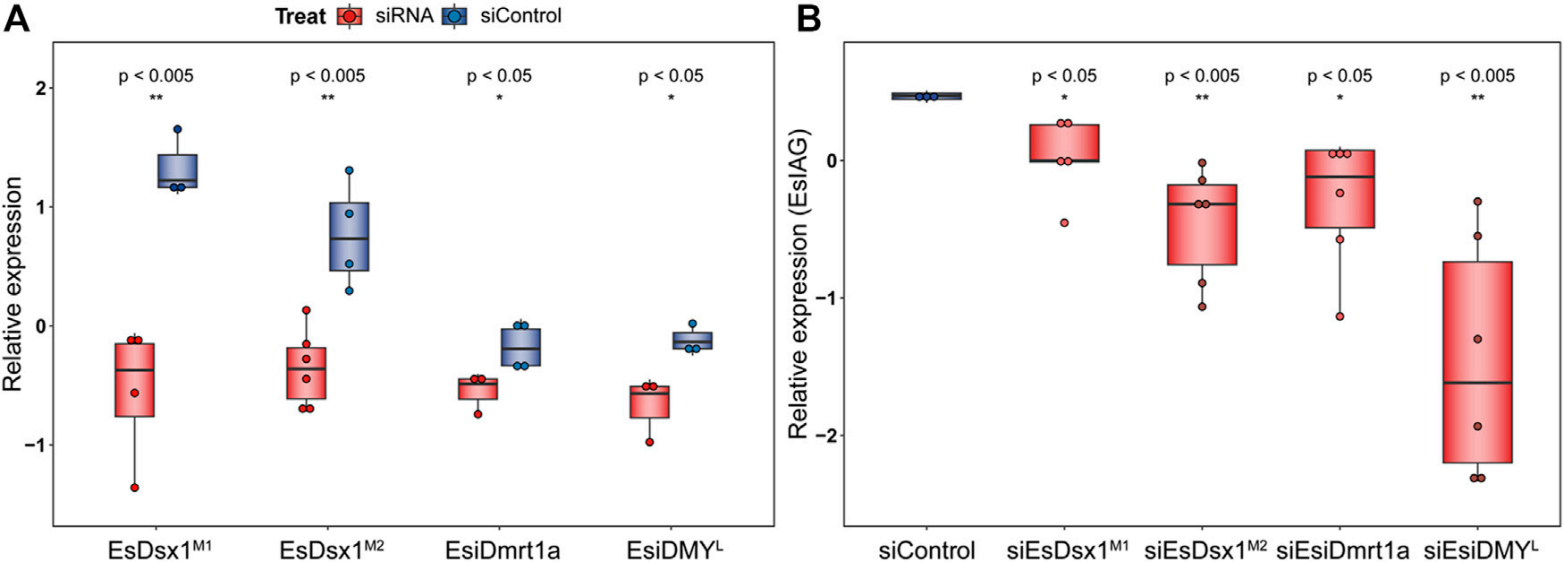
Function of *Dmrt* genes for *IAG* expression in *E. sinensis*. Transcript abundance was measured through RT-qPCR. Box plot is show as log-scale relative values of expression levels to the reference gene, *Esβ-actin*. Each dot represents the expression level of each individual. Independent *T*-test was performed to ascertain the difference between siRNA and siControl groups, with significance levels indicated (*p < 0.05, **p < 0.01) Statistical results are listed in the Supplementary Table S6. Expression levels of *EsDmrt* genes **(A)** and *EsIAG* gene **(B)** in RNAi and control groups are shown separately.

We conducted siRNA interference to investigate the role of the *EsDmrt* family in AG development, with *EsIAG* serving as a molecular marker. The results prove successful silencing of *EsDsx1* ^*M1*^, *EsDsx1* ^*M2*^, *EsiDmrt1a* and *EsiDMY* ^*L*^ genes (Figure 4A, independent sample *T-*test, *p* = 4.11E-03 in siEsDsx1^M1^ group, 2.00E-03 in siEsDsx1^M2^ group, 4.97E-02 in siEsiDmrt1a group, and 3.80E-02 in siEsiDMY^L^ group). Furthermore, all RNAi groups show a significant decrease in *EsIAG* expression (Figure 4B, independent sample *T-*test, *p* = 4.40E-02 in siEsDsx1^M1^ group, 3.00E-03 in siEsDsx1^M2^ group, 3.50E-02 in siEsiDmrt1a group and 3.00E-03 in siEsiDMY^L^ group).

### 3.6 Consensus analysis of DM domain in different *Dmrt* genes

**FIGURE 5:**
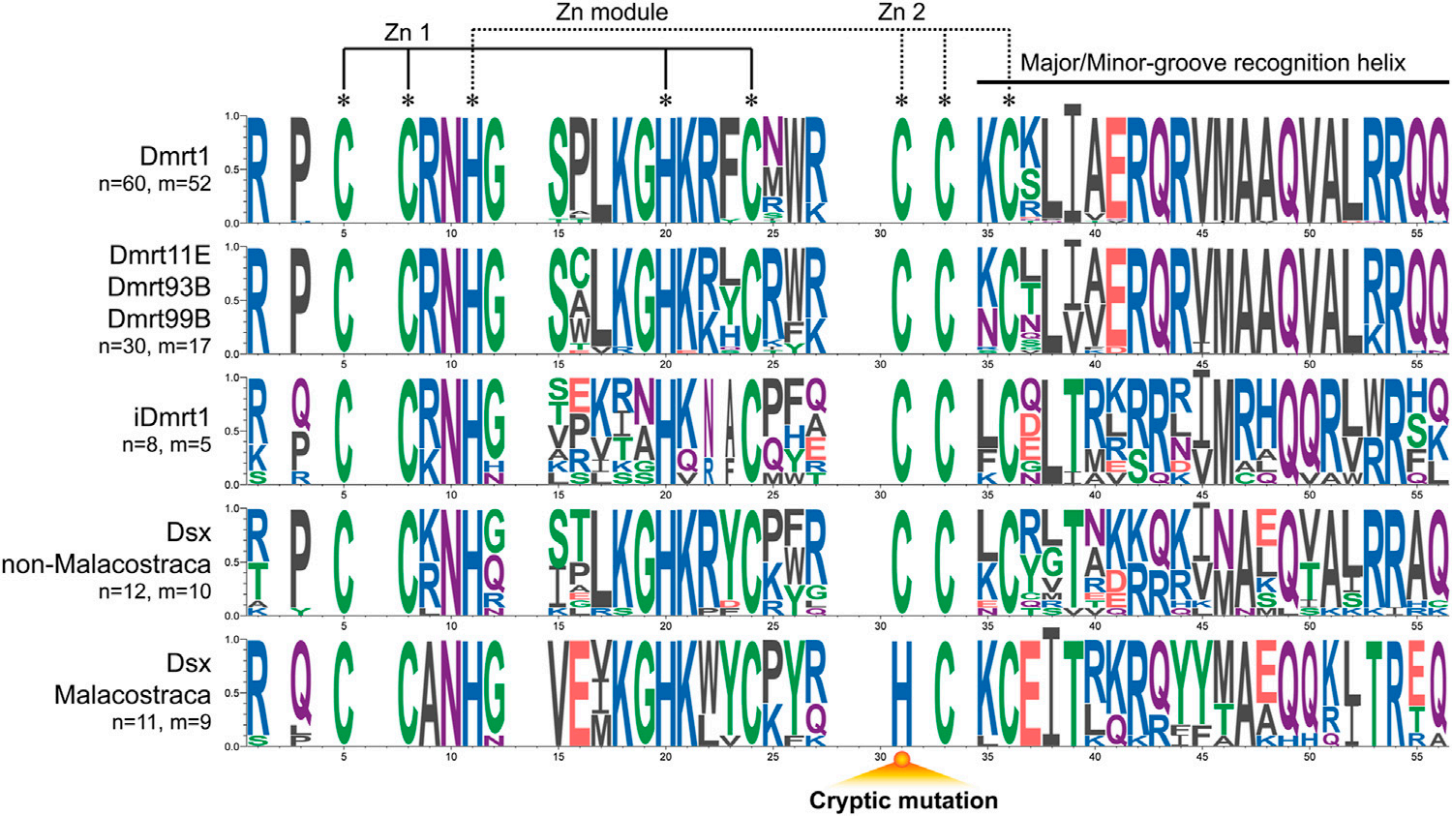
Consensus analysis of DM domains of the *Dmrt* gene family. Less conserved points are discarded. The cryptic mutation in Malacostraca *Dsx* is annotated below. Amino acids are colored according to their chemical properties: red (D, E) for acidic, green (C, G, S, T, Y) for polar, blue (H, K, R) for basic, black (A, F, I, L, M, V, W) for hydrophobic, and purple (Q, N) for neutral. n: number of unique sequences. m: number of different species.

In the analysis of the conservation of DM domains (Figure 5). We find that *Dsx* and *iDmrt1* groups exhibit great variability in the amino acids, which is different from the conservation observed in *Dmrt1*, *Dmrt11E*, *Dmrt93B* and *Dmrt99B* groups. The eight zinc motif-specific residues remain invariant in all tested *Dmrt* genes, except for the Malacostraca *Dsx*, where a cryptic mutation appears due to a substitution, from cysteine to histidine. Furthermore, there are some certain amino acids, including positions 9, 15, 16, 37, 38, and 53 that also distinguish the Malacostraca *Dsx* from other *Dmrt* genes.

### 3.7 Selection analysis of *iDmrt1* group

We used the site codon model in PAML to investigate ongoing selection pressure on the *iDmrt1* group. The selection test between M8 and M7 site models shows evidence of positive selection for the *iDmrt1* group (LRT, *p* = 0.0494, Table 1). The BEB analysis demonstrates that position 30 on the DM domain is under positive selection with a posterior probability greater than 0.95 (Table 1). Additionally, the comparison between M3 and M0 site models suggests variable *ω* values among different positions (LRT, *p* < 10^^−10^, Table 1).

**TABLE 1:** Analysis of positive selection using random site models on *iDmrt1* tree.

Site model	lnL	Site class	Proportion (p)	*ω*	Positively selected sites	LRT *p* value
M0	−1192.424219	0	NA	0.05351	NA	
M1a	−1159.966028	0	0.71966	0.28034	NA	
		1	0.03453	1		
M2a	−1159.966028	0	0.71966	0.03453	30	>0.99 (M2a vs. M1a)
		1	0.12545	1		
		2	0.15489	1		
M3	−1136.263954	0	0.28696	0	NA	<10^–10^ (M3 vs. M0)
		1	0.42242	0.0314		
		2	0.29062	0.23911		
M7	−1137.344442	0–9	0–9: 0.1	0.00003 0.00089 0.00409 0.01124 0.02424 0.04557 0.07903 0.13152 0.21904 0.40415	NA	
M8	−1134.337947	0–10	0–9: 0.09810, 10: 0.01903	0.00006 0.00108 0.00432 0.01089 0.02208 0.03960 0.06626 0.10733 0.17562 0.32419 5.26235	30	0.0494 (M8 vs. M7)

lnL, ln likelihood; LRT, likelihood ratio test; BEB, bayes empirical bayes.

### 3.8 Evolutionary history of *Dsx*

**FIGURE 6:**
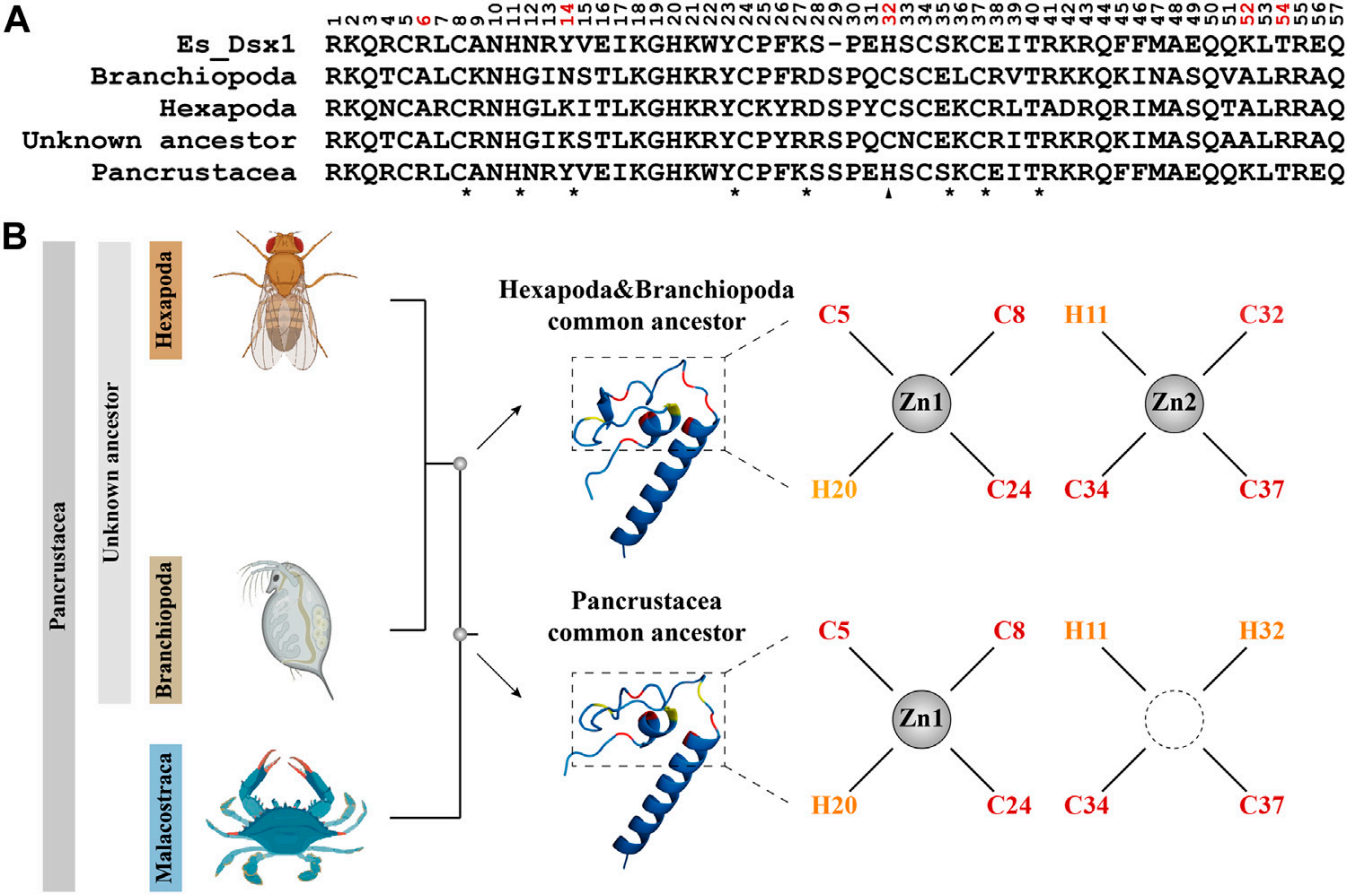
Ancestral sequence reconstruction of the DM domain from *Dsx* in Pancrustacea. **(A)** Alignment of the ancestral amino acid sequences of the DM domain from different common ancestor. Positions assumed to be positively selected are colored in red. Conserved residues are indicated with asterisks and mutation is indicated with a triangle. Es_Dsx1, *Dsx1* of *E. sinensis*, is shown as a reference. **(B)** Predicted protein structures of *Dsx* in different common ancestors. The phylogenetic relationship is based on topology from Rota-Stabelli et al. (2013). 3D images in the midst indicate modelled proteins of DM domains with the motif-specific residues highlighted in red (cysteine) and yellow (histidine). Chelation of zinc atoms is visualized as a result of ZincExplore.

The LRT result shows that Model A fits the reference tree significantly better than the Model Null (LRT, *p* = 0.046384445, Table 2), indicating selection pressure acting on the foreground branch. The BEB analysis demonstrates that five positions on the DM domain, including the cryptic mutation shown in Figure 5, are under positive selection. Ancestral sequence reconstruction reveals that the cryptic mutation is also present in the common ancestor of Pancrustacea, but not in the common ancestor of Hexapoda and Branchiopoda (Figure 6A). According to the prediction of ZincExplorer (Supplementary Figure S6), the *Dsx* protein in the common ancestor of Pancrustacea could bind only one zinc atom (Figure 6B), which is consistent with the three-dimension structures of Malacostraca *Dsx* modelled using Swiss-Model (Supplementary Figure S7).

**TABLE 2:** Analysis of positive selection using branch-site models on *Dsx* tree.

Model	lnL	Site class	Proportion	Background *ω*	Foreground *ω*	Positively selected sites	LRT *p* value
A	−7704.554096	0	0.51052	0.04305	0.04305	6, 14, 32, 52, 54	
		1	0.10762	1	1		
		2a	0.31537	0.04305	999		
		2b	0.06648	1	999		
Null	−7706.537885	0	0.53548	0.04193	0.04193		0.046384445
		1	0.11237	1	1		
		2a	0.29107	0.04193	1		
		2b	0.06108	1	1		

lnL, ln likelihood; LRT, likelihood ratio test; BEB, bayes empirical bayes.

## 4 Discussion

In our previous studies, we have confirmed the names of *EsDmrt11E* (Du et al., 2019) and *EsiDMY* (Cui et al., 2021). *EsDmrt-like* and *EsDsx-like* were reported in Zhang and Qiu (2010) and Wang et al. (2023), respectively. In this study, we rename three genes previously identified in Du et al. (2019) based on their phylogenetic relationships, which are *EsDmrt93B*, *EsiDmrt1a* and *EsiDmrt1b* (formerly known as *EsDmrt3*, *EsIdmrt1-1* and *EsDsx2*). Furthermore, we find the duplicated *Dsx* genes, *EsDsx1* and *EsDsx2*, which form a tandem gene cluster on the genome. As a result, we identify a total of nine members of the *Dmrt* family in the crab.

In invertebrates, *Dmrt* genes are known to play a crucial role in sexual differentiation, driving sex-specific development in varied tissues and cell types (Matson and Zarkower, 2012). In crustaceans, *Dmrt* genes have been found to transcribe in a strongly male-biased way, indicating their specific involvement in male morphogenesis (Wexler et al., 2019). Our transcriptomic analyses of the *Dmrt* family in *E. sinensis* support and expand on previous findings, suggesting that the *EsDmrt* family primarily functions in male sexual differentiation, specifically promoting the development of testis and AG. Previous studies have meticulously described the function of *EsDmrt-like* (Zhang and Qiu, 2010) and *EsDsx-like* (Wang et al., 2023) during spermatogenesis. In our research, we find the abundant expression of *EsDsx1*, *EsDsx2*, *EsiDmrt1a* and *EsiDMY* in AG, as well as the aberrant transcription of *EsiDmrt1a* and *EsiDMY* in the chimeric AG. Furthermore, RNA knockdown on the transcripts from *EsDsx1*, *EsiDmrt1a* and *EsiDMY* could result in a significant decrease in *EsIAG* transcription. Taken together, our findings propose that in *E. sinensis*, both genes from *Dsx* and *iDmrt1* groups may function in AG development.

In Malacostraca, *Dsx* genes have been extensively studied and are known to upregulate *IAG* in various species, such as *F. chinensis* (Li et al., 2018), *S. paramamosain* (Wan et al., 2022) and *P. monodon* (Wei et al., 2022). And *iDmrt1* genes has also showed closed relationship with AG in species such as *M. rosenbergii* (Abayed et al., 2019), where the *MroiDmrt1b* and *MroiDmrt1c* genes are highly enriched in AG, and *Portunus trituberculatus*, where the *PtiDmrt1* gene upregulates the *PtIAG* expression (Wang et al., 2020). These findings suggest that the involvement of both *Dsx* and *iDmrt1* genes in AG development might be a common feature of Malacostraca.

Our phylogenetic tree showed that the *Dsx* and *iDmrt1* genes in Malacostraca form two distinct monophyletic clades. On the one hand, the *iDmrt1* gene was first identified in the *S. verreauxi* (Ventura et al., 2020), and subsequently identified solely in malacostracan species. Our selection analysis (Table 1) reveals that the *iDmrt1* group is subjected to positive selection, suggesting a unique function that is specific to the Malacostraca, i.e., promoting AG development. On the other hand, the Malacostraca *Dsx* shows unique amino acid residues at position 9, 38 and 53 (Figure 5). These positions have been confirmed to play critical roles in DNA binding and homodimerization (Murphy et al., 2015). Moreover, and perhaps more significantly, an invariant cysteine residue in the zinc binding module, known to coordinate with zinc atom (Zhu et al., 2000), is replaced with a histidine only in the Malacostraca *Dsx* (Figure 5). Therefore, we suggest that the Malacostraca *Dsx* may not bind to DNA directly but instead form heterodimers with the *iDmrt1*. Such binding mode also rationalizes their co-expression in AG (Figure 2B), which is similar to the situation observed in vertebrates, where *Dmrt3* heterodimerizes with the co-expressed *Dmrt1* in testis (Murphy et al., 2007).

**FIGURE 7:**
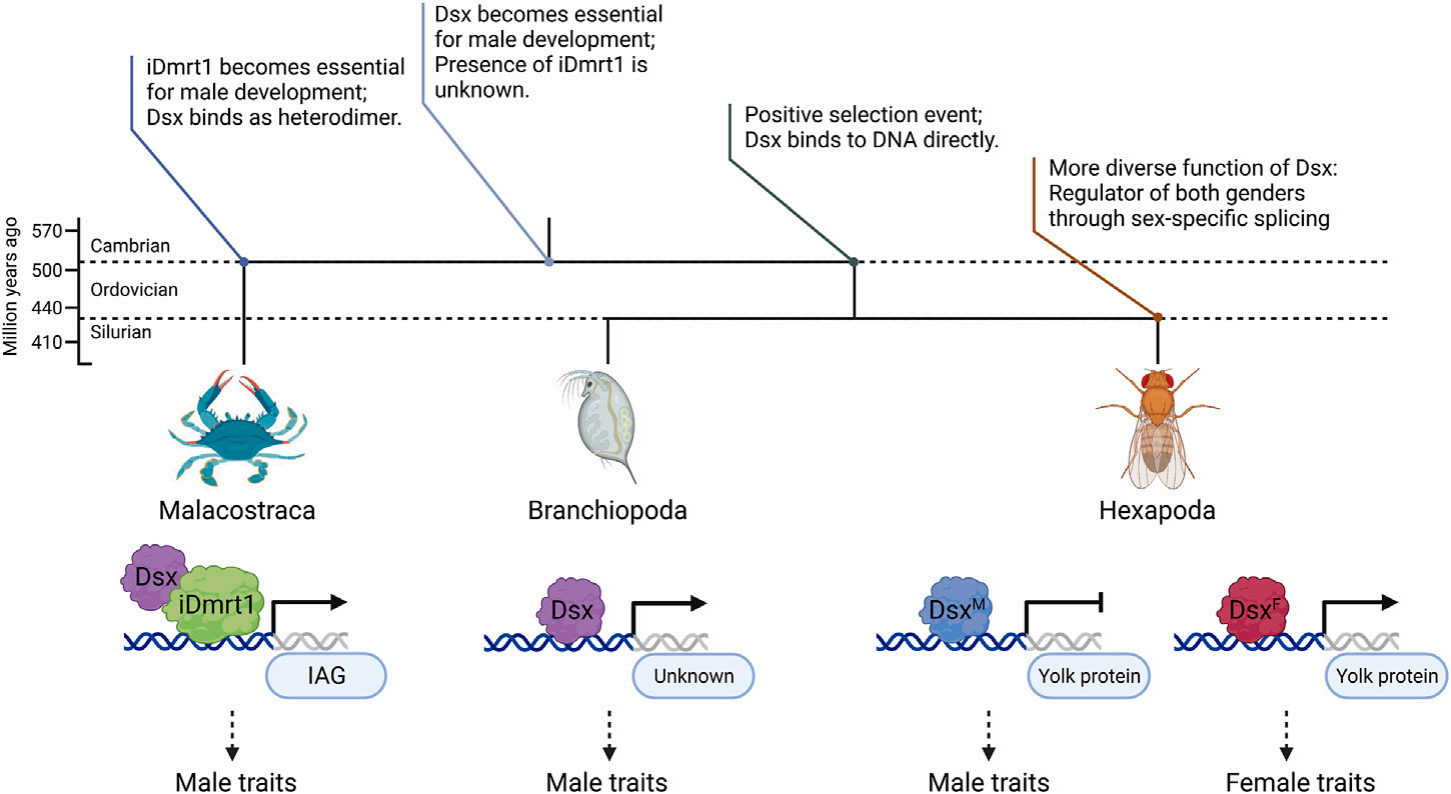
Diagram of the evolutionary history of *Dsx* and *iDmrt1* proposed in this study. The upper topological structure and annotations describe possible statuses of *Dmrt* in different common ancestors. The lower part compares the transcriptional regulation of *Dsx* in Malacostraca, Hexapoda and Branchiopoda. Previous research has suggested that heterodimer formation of *Dmrt* proteins may contribute to transcriptional regulation (Murphy et al., 2007). Mutations in the DM domain and the lack of an oligomerization domain (Zheng et al., 2020) suggest that Malacostraca *Dsx* may not directly bind to, but rather heterodimerize on DNA with another *Dmrt* protein, which, according to our evolutionary analysis, could be *iDmrt1*. Figure created using BioRender.

Selection analysis (Table 2) and ancestral sequence reconstruction (Figure 6) place Malacostraca *Dsx* as an outgroup to Hexapoda and Branchiopoda, which is consistent with previous studies on *Hox* (Averof and Akam, 1995) and *elongation factor-1 alpha* (Regier and shultz, 1997) genes. Based on these findings, we incorporated Malacostraca into the stepwise evolutionary history proposed by Wexler et al. (2019) and Chikami et al. (2022) (Figure 7) that *Dsx* has become essential for male development in the common ancestor of Pancrustacea; then during the split of Malacostraca and the common ancestor of Branchiopoda and Hexapoda in the late Cambrian, a selective event, which might result from ecological or physiological differences (Glenner et al., 2006), either builds or reserves the unique regulatory mode of *Dmrt* genes in Malacostraca.

In this study, we investigated the *Dmrt* family in a decapod crab and explored its function in AG development, which extend our knowledge of sexual development mechanisms in this species. Besides, we proposed a hypothesis of transcriptional regulation based on the finding of the intriguing *Dsx* and *iDmrt1* groups in Malacostraca. Further investigation would be necessary to test our hypothesis and unravel the evolutionary history of DM domain genes.

## Data Availability

The datasets presented in this study can be found in online repositories. The names of the repository/repositories and accession number(s) can be found in the article/Supplementary Material.
