# Supplementary material for: Analyses of the Dmrt family in a decapod crab, Eriocheir sinensis uncover new facets on the evolution of DM domain genes
Source: Front Physiol. 2023 May 26;14:1201846. doi: 10.3389/fphys.2023.1201846 (PMC10252143; doi:10.3389/fphys.2023.1201846)
Supplement: Supplementary file 2 [file Table2.docx]

Supplementary Material

**Analyses of the *Dmrt* family in a decapod crab, *Eriocheir sinensis* uncover new facets on the evolution of DM domain genes**

**Peng Zhang, Yanan Yang, Yuanfeng Xu, Zhaoxia Cui^*^**

*** Correspondence:** Corresponding Author: cuizhaoxia@nbu.edu.cn

# Supplementary Figures

**
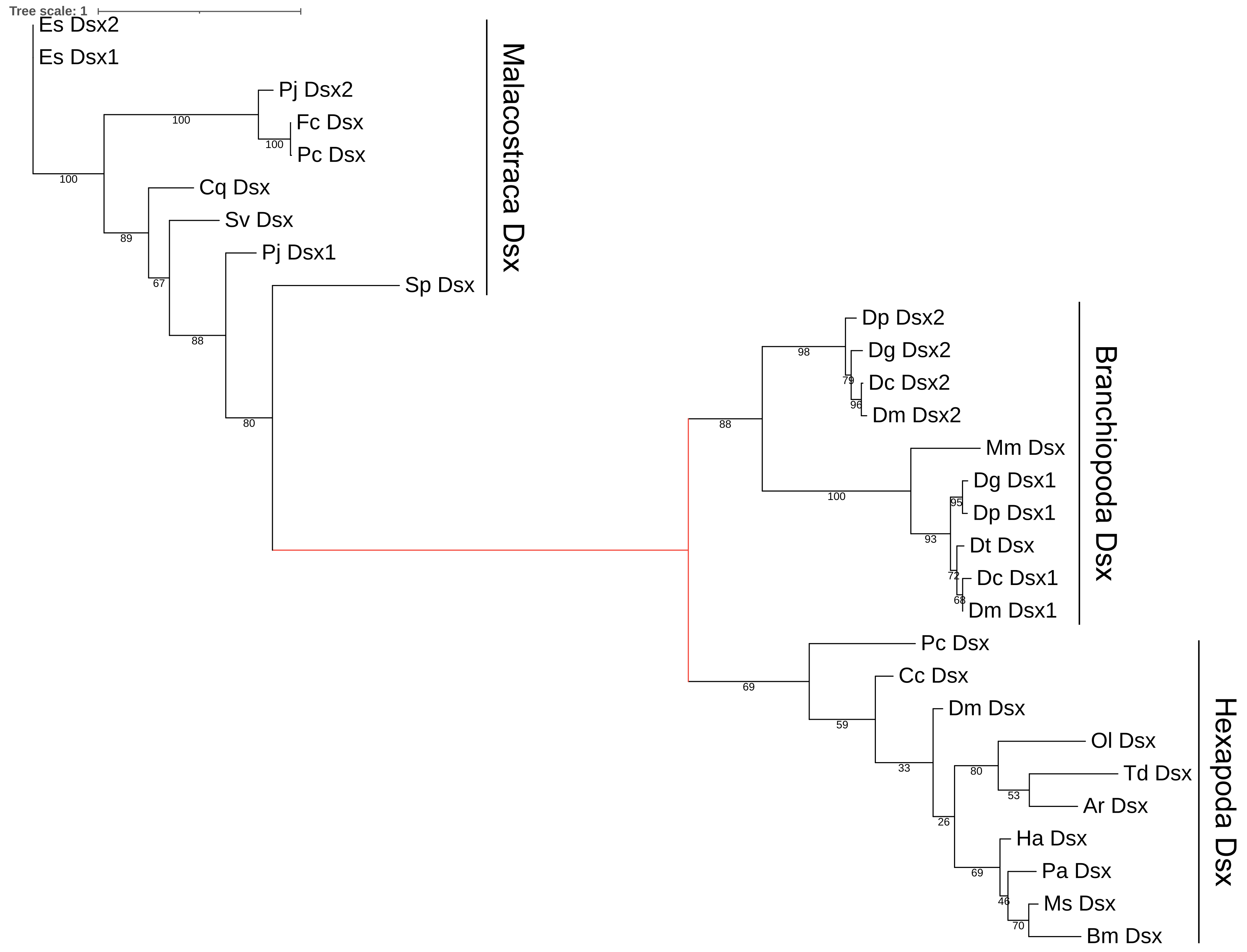
**

**Supplementary Figure 1.** The guide tree used for positive selection analysis and ancestral sequence reconstruction of the *Dsx* groups in Pancrustacean. The foreground branch was annotated as red. The tree was constructed using the nucleotide alignment of the N-terminus of *Dsx*, including the N-terminal DM domain, by PRANK and IQ-TREE.


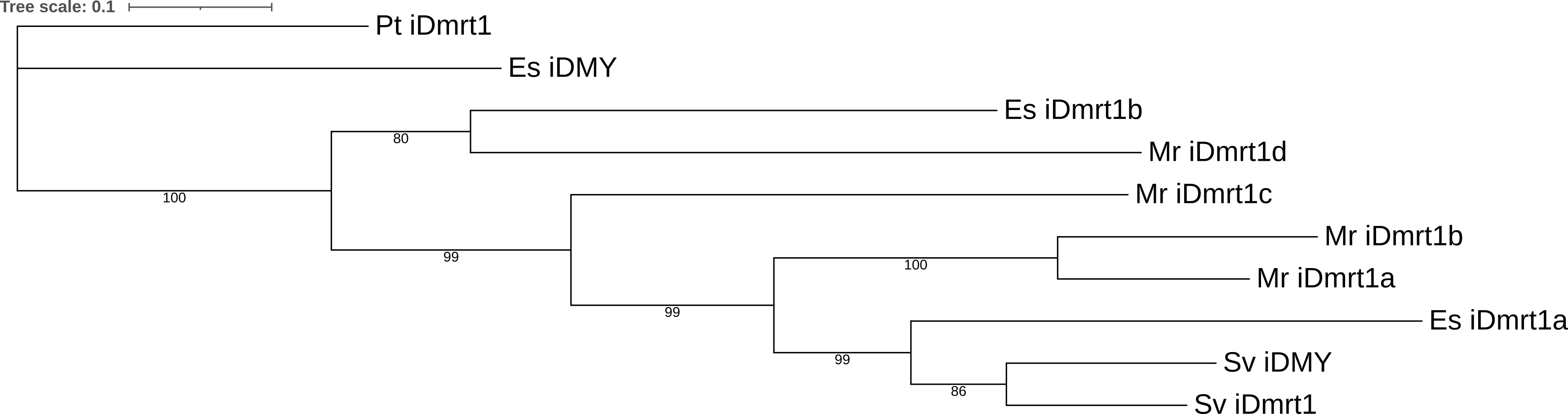


**Supplementary Figure 2.** The guide tree used for positive selection analysis of the *iDmrt1* group in Malacostraca. The tree was constructed using the nucleotide alignment of the N-terminus of *iDmrt1*, including the N-terminal DM domain, by PRANK and IQ-TREE.


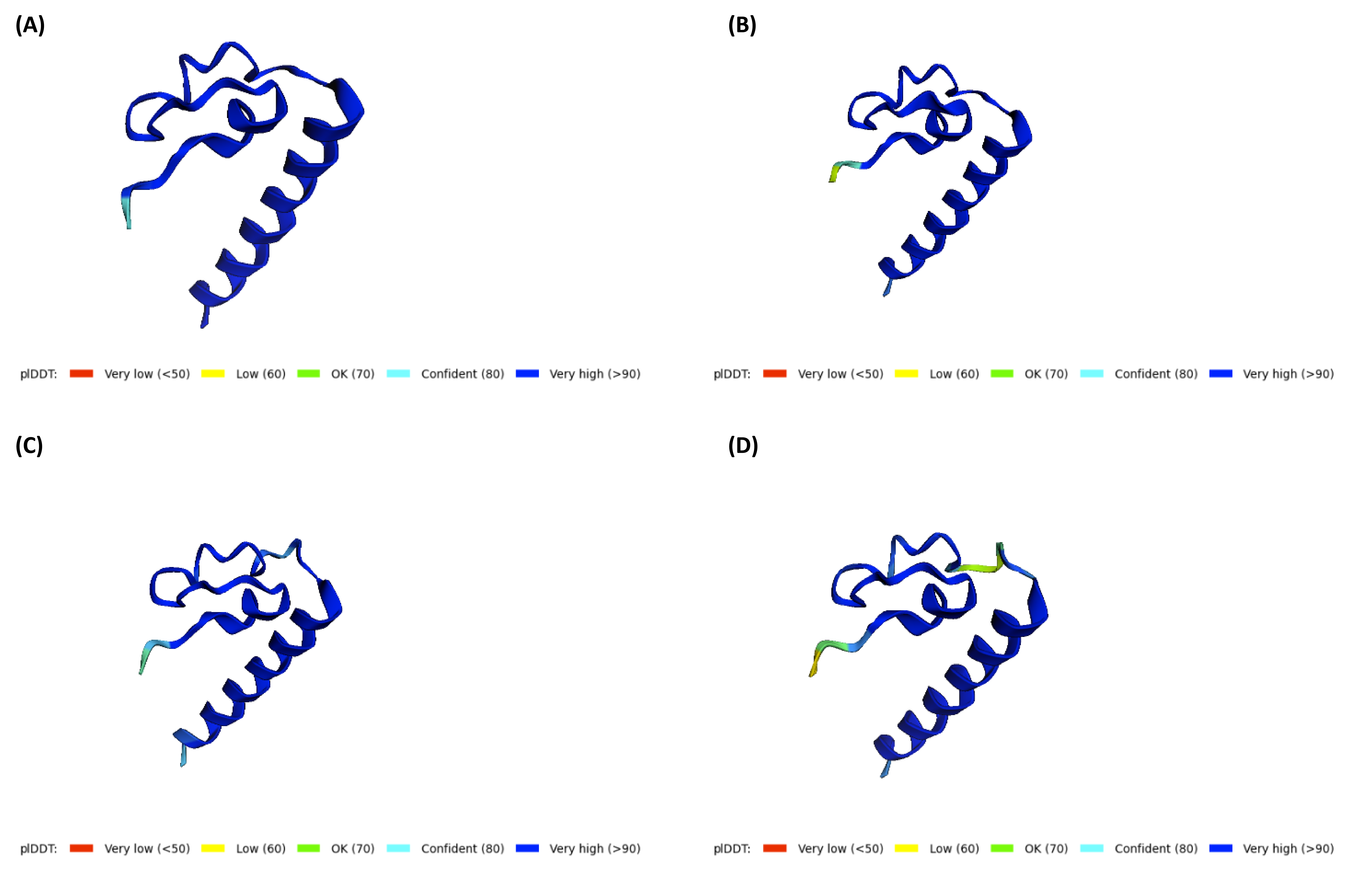


**Supplementary Figure 3.** Accuracy of structure predictions of the DM domain of Dsx. Results of the prediction of DM structure of Dsx in common ancestor of Branchiopoda. **(A)** the common ancestor of Hexapoda. **(B)** the common ancestor of Branchiopoda and Hexapoda. **(C)** and the common ancestor of Pancrustacea. **(D)** In each panel, the right 3D model shows the predicted structure of *Dsx* colored by its predicted local distance difference test (plDDT) score. The legend of color in the 3D model is shown at the bottom of the figure.


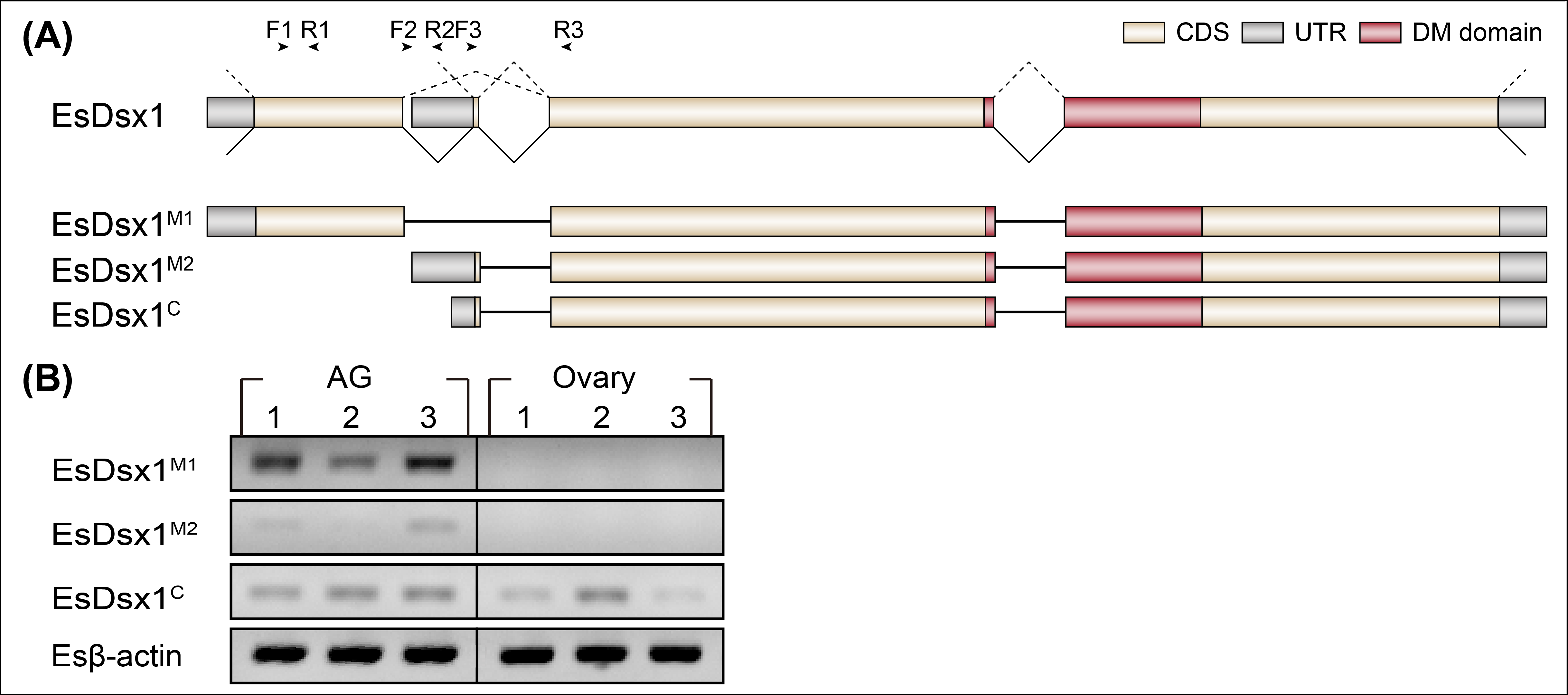


**Supplementary Figure 4.** Identification of alternative splicing events in *EsDsx1*. **(A)** Diagram of putative *EsDsx1* pre-mRNAs. *EsDsx1^M1^* and *EsDsx1^M2^* encode for two different proteins. *EsDsx1^C^* and *EsDsx1^M2^* encode the same protein and only differ in the 5’ UTR. Arrows showed primer positions. Primer F1 and R1 were used to amplify partial sequence of *EsDsx1^M1^*, primer F2 and R2 were used to amplify partial sequence of *EsDsx1^M2^*, and primer F3 and R3 were used to amplify partial sequence of *EsDsx1^C^* (Primer sequences are listed in Supplementary Table 5). **(B)** Gel picture showing two isoforms that expressed specifically in AG compared to ovary, and one isoform that expressed in both. *Esβ-actin* was conducted as a reference. M represents DNA size marker.


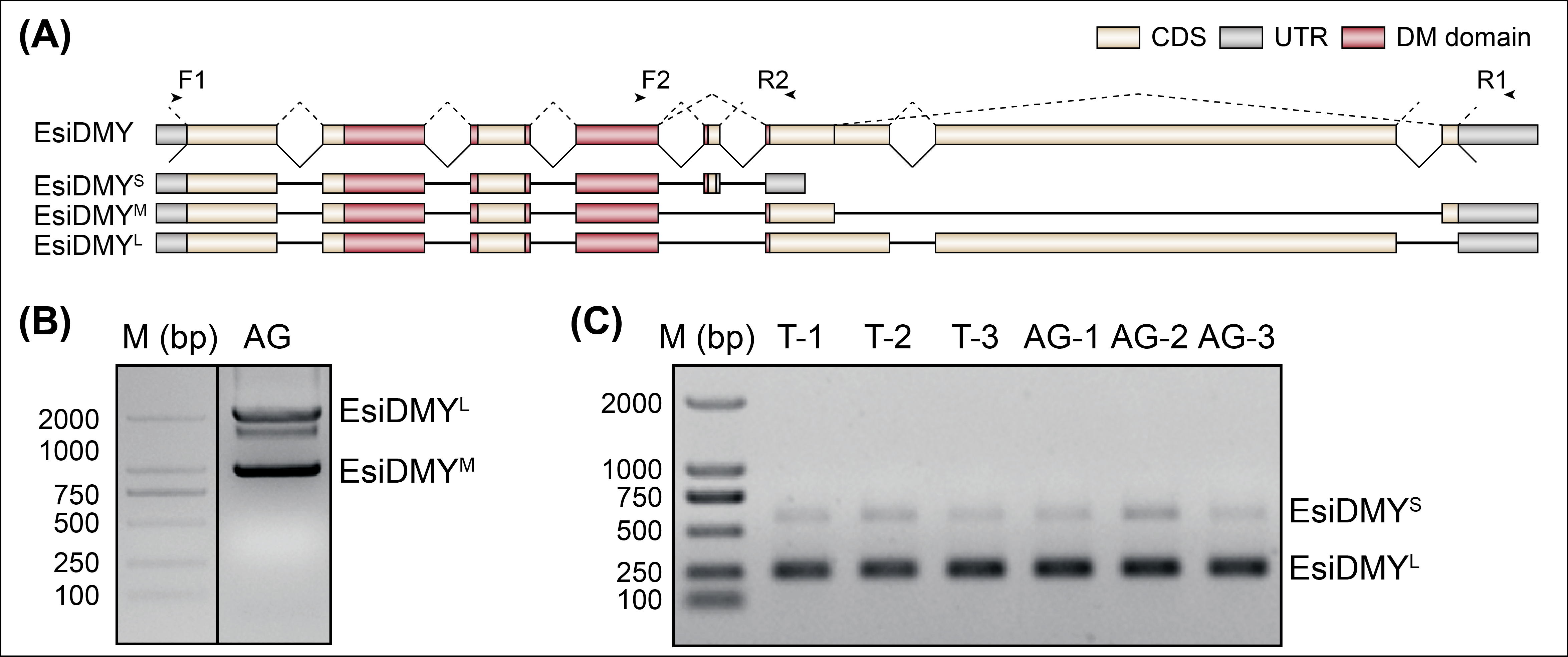
**Supplementary Figure 5.** Identification of alternative splicing events in *EsiDMY*. **(A)** Diagram of putative *EsiDMY* pre-mRNAs. *EsiDMY^S^*, *EsiDMY^M^* and *EsiDMY^L^* encode for three different proteins. Arrows showed primer positions. Primer F1 and R1 were used to amplify full ORF of *EsiDMY^L^* and *EsiDMY^M^*. Primer F2 and R2 were used to detect alternative splicing event of exon skipping (Primer sequences are listed in Supplementary Table 5). **(B)** Gel picture showing two bands (*EsiDMY^L^* and *EsiDMY^M^*) in AG and one band (uncharacterized) as a result of RT-PCR using primer 1. M represents DNA size marker. **(C)** Gel picture showing two bands (*EsiDMY^L^* and *EsiDMY^S^*) in testis and AG as a result of RT-PCR using primer 2. M represents DNA size marker.





**Supplementary Figure 6.** Column graph illustrating zinc binding prediction score for DM domain from ancestral sequences using ZincExplorer. The mutation that causes the loss of one zinc ion is indicated with blue dot.


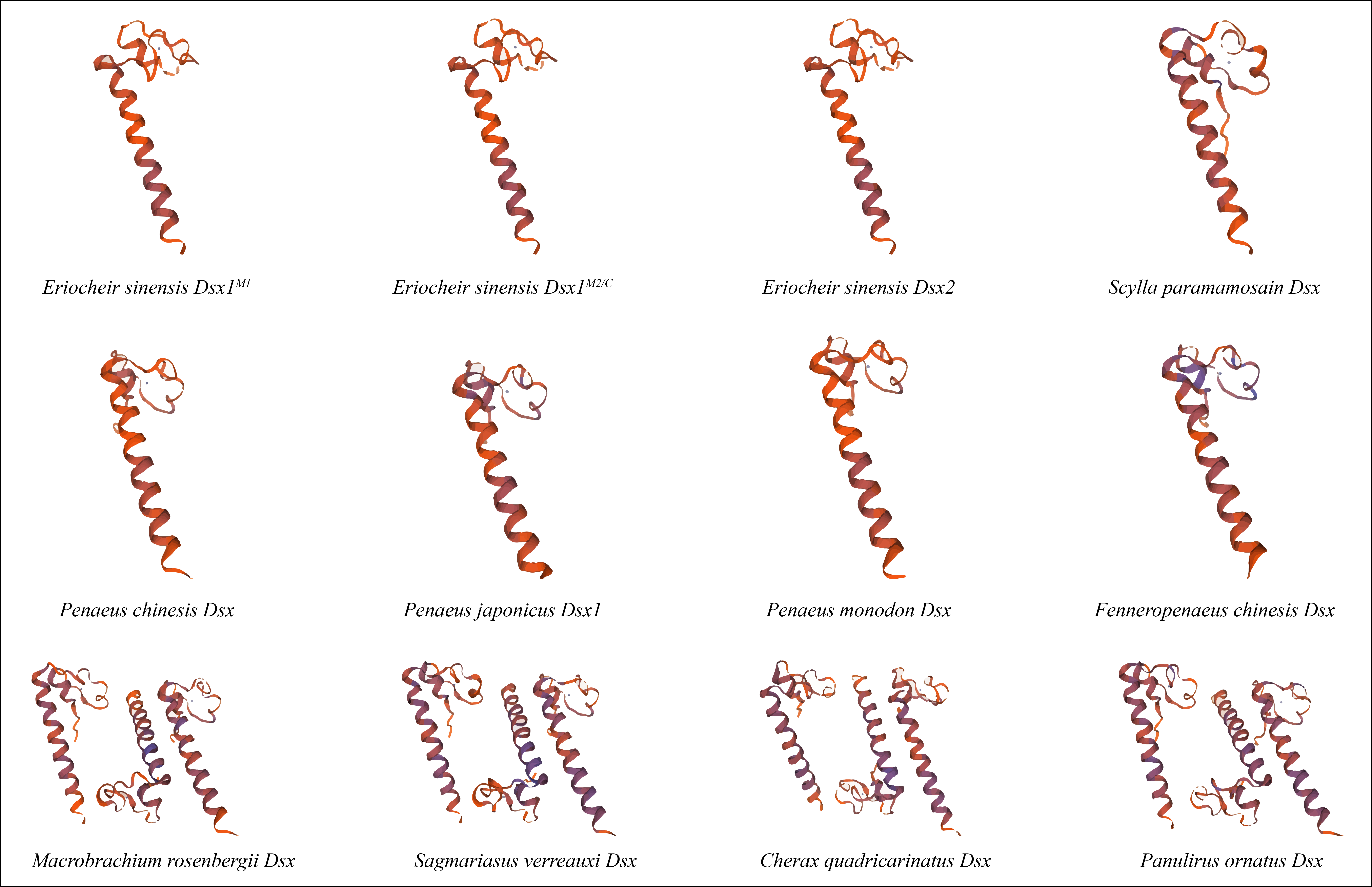


**Supplementary Figure 7.** Three-dimension structures of Malacostraca Dsx modelled using Swiss-Model online software. Each predicted protein only chelates one zinc ion.
